# Das Zweitmeinungsverfahren in der Elektrophysiologie: Rechtliche Aspekte für Behandler:innen und Zweitmeiner:innen

**DOI:** 10.1007/s00399-023-00951-6

**Published:** 2023-07-14

**Authors:** Eva Rütz, Denise Bartels, Julia Vogler, Roland R. Tilz

**Affiliations:** 1 Luther Rechtsanwaltsgesellschaft mbH, Graf-Adolf-Platz 15, 40213 Düsseldorf, Deutschland; 2 Klinik für Rhythmologie, Universitäres Herzzentrum Lübeck Universitätsklinikum Schleswig-Holstein (UKSH), Ratzeburger Allee 160, 23538 Lübeck, Deutschland; 3 German Center for Cardiovascular Research (DZHK), Partner Site Lübeck, Lübeck, Deutschland

**Keywords:** Zweitmeinungsverfahren, Aufklärung, Zweitmeiner, Mengenanfällige planbare Eingriffe, Sachleistung, Referral and consultation, Informed consent, Second opinion provider, Scheduled procedures, Non-cash service

## Abstract

Gesetzlich versicherte Patient:innen haben die Möglichkeit, vor Durchführung bestimmter Prozeduren zulasten ihrer Krankenversicherung eine zweite Meinung neben der ihres primären Behandlers einzuholen. Auf Seiten der Behandler:innen wie auch der Zweitmeiner:innen entstehen hierdurch verschiedene rechtliche Pflichten und Fragestellungen. Dieser Beitrag erläutert in Grundzügen, welche Pflichten jeweils für Behandler:innen und Zweitmeiner:innen bestehen und wer als Zweitmeiner:in geeignet ist. Ein besonderer Fokus liegt hierbei zum einen auf der Aufklärung über die Möglichkeit der Einholung einer Zweitmeinung durch den/die Behandler:innen. Zum anderen werden die Anforderungen an die Zweitmeiner:innen und deren Aufgaben dargestellt.

## Abstract (en)

Patients with statutory health insurance in Germany have the right to obtain a second opinion at the expense of their health insurance in addition to that of their primary practitioner before certain procedures are carried out. This gives rise to various legal obligations and questions on the part of both the practitioner and the second opinion provider. This article outlines, based on the legal situation, which obligations exist for both practitioners and second opinion providers, and who is suitable to provide a second opinion. Focus is placed on informing patients about the possibility of obtaining a second opinion. On the other hand, it explains which requirements second opinion providers have to fulfill and which duties are incumbent on them.

Das Zweitmeinungsverfahren nach § 27b Fünftes Buch Sozialgesetzbuch (SGB V) wurde mit dem Gesetz zur Stärkung der Versorgung in der gesetzlichen Krankenversicherung in den Umfang der dem gesetzlich Versicherten zustehenden Leistungen aufgenommen. Hintergrund war die Befürchtung des Gesetzgebers, dass bei bestimmten Eingriffen aus wirtschaftlichen Gründen eine medizinisch nicht notwendige Ausweitung der Indikationsstellung erfolgen könnte [1]. Die Festlegung des Kreises der Eingriffe, für die der Anspruch auf Einholung einer Zweitmeinung besteht, wurde dem Gemeinsamen Bundesausschuss (G-BA) auferlegt [1].

## Anspruch gesetzlich Versicherter auf Zweitmeinung

§ 27b SGB V schafft einen Anspruch für gesetzlich versicherte Personen auf Einholung einer Zweitmeinung auf Kosten ihrer gesetzlichen Krankenversicherung und verpflichtet daher auch Letztere. Sie ist gemäß § 73 Abs. 2 Satz 1 Nr. 13 SGB V Bestandteil der vertragsärztlichen Versorgung. Zusätzlich enthält die Norm aber auch Pflichten für Zweitmeiner:innen (s. unten). Der Anspruch der gesetzlich Versicherten ist beschränkt auf bestimmte, durch den G‑BA zu bestimmende Eingriffe (§ 27b Abs. 2 SGB V). Die Krankenkassen können zudem in ihren Satzungen zusätzliche Leistungen für die Einholung einer Zweitmeinung vorsehen (§ 27b Abs. 6 SGB V).

Eine Zweitmeinung ist in § 2 des Allgemeinen Teils der Richtlinie des G‑BA zum Zweitmeinungsverfahren (Zm-RL; [2]) definiert als „unabhängige, neutrale ärztliche zweite Meinung bei einem Leistungserbringer nach § 27b Absatz 3 SGB V zu den im Besonderen Teil dieser Richtlinie benannten planbaren Eingriffen“. Gegenstand des Zweitmeinungsverfahrens ist die Indikationsstellung (s. unten). Die Überweisung von Patient:innen an andere Ärzt:innen zwecks Prüfung, ob die Indikation zu einem der einschlägigen Eingriffe besteht, ist demnach nach Ansicht der Verfasser:innen noch keine Erstmeinung, da noch keine Indikation gestellt wurde.

Mit Beschluss vom 18. März 2022, der am 31. Mai 2022 in Kraft getreten ist, hat der G‑BA mit den kathetergestützten elektrophysiologischen Herzuntersuchungen und Ablationen am Herzen einen elektrophysiologischen Eingriff in die Gruppe derjenigen Eingriffe aufgenommen, die für das Zweitmeinungsverfahren vorgesehen sind. Gegenstand des Zweitmeinungsverfahrens ist gemäß § 1 Abs. 2 zu Eingriff 7 im Besonderen Teil Zm-RL die *Indikationsstellung* zu dem benannten Eingriff. Der Eingriff ist unabhängig von der jeweiligen Grunderkrankung erfasst; ausgeschlossen sind Notfalleingriffe und dringliche Eingriffe (§ 1 Abs. 2 Eingriff 7 Besonderer Teil Zm-RL) (Tab. 1).

**Table 1:** 

Eingriff 7 Besonderer Teil Zm-RL	Kathetergestützte elektrophysiologische Herzuntersuchungen und Ablationen am Herzen	„Der Eingriff umfasst kathetergestützte elektrophysiologische Herzuntersuchungen und Ablationen am Herzen unabhängig von der jeweiligen Grunderkrankung. Nicht umfasst sind Notfalleingriffe und dringliche Eingriffe.“
Eingriff 8 Besonderer Teil Zm-RL	Implantation eines Herzschrittmachers, eines Defibrillators oder eines CRT-Aggregats	„Der Eingriff umfasst die Implantation eines Herzschrittmachers oder eines Defibrillators (Herzschrittmacher, ICD-, CRT-P- und CRT-D-Aggregate) unabhängig von der jeweiligen Grunderkrankung. Nicht erfasst sind Notfalleingriffe, dringliche Eingriffe sowie Eingriffe zum Wechsel von Geräten allein aufgrund von Batterieermüdung ohne Systemwechsel.“

Mit Beschluss vom 19. Mai 2022 (Inkrafttreten am 28. Juli 2022) wurden zudem Eingriffe zur Implantation eines Herzschrittmachers oder eines Defibrillators aufgenommen. Auch hier ist – unabhängig von der jeweiligen Grunderkrankung – die Stellung der Indikation zum Eingriff Gegenstand des Zweitmeinungsverfahrens (§ 1 Abs. 1 Eingriff 8 Besonderer Teil Zm-RL). Nicht erfasst sind „Notfalleingriffe, dringliche Eingriffe sowie Eingriffe zum Wechsel von Geräten allein aufgrund von Batterieermüdung ohne Systemwechsel“.

## Aufgaben der indikationsstellenden Ärzt:innen, insbesondere Aufklärung

Im Zweitmeinungsverfahren treffen die indikationsstellenden Ärzt:innen vier besondere Pflichten.

1. Sie haben Patient:innen auf Informationsangebote über Zweitmeiner:innen *hinzuweisen* (§ 6 Abs. 2 Allgemeiner Teil Zm-RL). Hierbei sind Patient:innen auch darauf hinzuweisen, dass die Zweitmeinung bei *unabhängigen Ärzt:innen* einzuholen ist (s. unten). Den indikationsstellenden Ärzt:innen wird hierdurch eine *Informationspflicht* übertragen, die eigentlich den Krankenkassen obliegt, da diese grundsätzlich für die Information ihrer Versicherten verantwortlich sind [7]. Ein bundesweites Verzeichnis der Ärzt:innen, die über eine Genehmigung zur Erbringung von Zweitmeinungen verfügen, findet sich online über die Arztsuche im Bereitschaftsdienstportal der Kassenärztlichen Vereinigungen (www.116117.de/zweitmeinung).
2. Indikationsstellende Ärzt:innen haben zudem auf das „*Patientenmerkblatt*“ des G‑BA *hinzuweisen* (§ 6 Abs. 3 Allgemeiner Teil Zm-RL).
3. Patient:innen sind weiterhin darauf aufmerksam zu machen, dass sie gemäß § 630g Abs. 2 Bürgerliches Gesetzbuch (BGB) berechtigt sind, *Einsicht* in die sie betreffenden *Behandlungsunterlagen* zu nehmen. Die Kosten für das Zusammenstellen und Überlassen der für die Zweitmeinung maßgeblichen Behandlungsunterlagen tragen, anders als bei der Aushändigung von Behandlungsunterlagen üblich [3], nicht die Patient:innen selbst (vgl. § 630g Abs. 2 Satz 2 BGB), sondern die jeweilige Krankenkasse (§ 6 Abs. 4 Allgemeiner Teil Zm-RL). Dieser Hinweis soll den Patient:innen ermöglichen, ihre Behandlungsunterlagen der indikationsstellenden Ärzt:innen zur Grundlage der Zweitmeinung zu machen, so dass Untersuchungen nicht durch die Zweitmeiner:innen wiederholt werden müssen [4].
4. Die *wichtigste Pflicht* für indikationsstellende Ärzt:innen ist die *Aufklärung* der gesetzlich versicherten Patient:innen über das *Recht zur Einholung einer Zweitmeinung* bzgl. der betroffenen Eingriffe (vgl. § 27b Abs. 5 Satz 1 SGB V). Die Aufklärung muss *mündlich* erfolgen und mindestens *10 Tage vor dem geplanten Eingriff*, jedenfalls aber so rechtzeitig stattfinden, dass die Versicherten ihre Entscheidung zur Einholung einer Zweitmeinung nach reiflicher Überlegung treffen können (§ 27b Abs. 5 Satz 2, 3 und 4 SGB V). Es ist empfehlenswert, im Regelfall die explizit gesetzlich geregelte zehntägige Frist auch einzuhalten, in jedem Fall aber sorgfältig zu dokumentieren, dass Patient:innen eine ausreichende Bedenkzeit eingeräumt wird. Ein regelmäßiger schriftlich gefasster Verzicht auf die zehntägige Frist ist nicht anzuraten. Die Patient:innen sollen bei lebensnaher Betrachtung Gelegenheit haben, eine Zweitmeinung einzuholen.
  Hintergrund dieser maßgeblichen Pflicht der indikationsstellenden Ärztinnen ist, dass die vom Zweitmeinungsverfahren erfassten Eingriffe sämtlich als *mengenanfällige* Eingriffe betrachtet werden, also hier die Gefahr vermutet wird, dass aus wirtschaftlichen statt aus rein medizinischen Gründen die Indikation zu einem solchen Eingriff gestellt wird [5]. Hiervor soll das Zweitmeinungsverfahren insbesondere gesetzlich versicherte Patient:innen schützen [1]. Die Aufklärung über die Zweitmeinung gleicht in Aufbau und Modalitäten der zivilrechtlichen Aufklärung aus dem Recht des Behandlungsvertrages gemäß § 630e BGB [1, 6]. Auch bei der zivilrechtlichen Aufklärung finden sich Verpflichtungen zur Mündlichkeit und zu der Frage des angemessenen Zeitrahmens.
  *Rechtlich nicht geklärt* sind allerdings die *haftungsrechtlichen Folgen eines Verstoßes gegen die Pflicht zur Aufklärung über das Zweitmeinungsverfahren*. Gemäß § 630d Abs. 2 BGB ist Voraussetzung für eine wirksame Einwilligung von Patient:innen in einen Eingriff die Aufklärung über alle für die Einwilligung maßgeblichen Umstände nach § 630e Abs. 1 bis 4 BGB [9].
  *Teilweise* wird vor diesem Hintergrund vertreten, dass die Pflicht zur Aufklärung über den Anspruch auf Zweitmeinung eine *weitere Aufklärungsverpflichtung* im Sinne des § 630e BGB darstelle. Dies würde bedeuten, dass eine fehlerhafte oder nicht durchgeführte Zweitmeinungsaufklärung einen Verstoß gegen das Selbstbestimmungsrecht der Patient:innen darstellte und so zu einem Haftungsrisiko führen könnte. Die Patient:innen hätten nicht wirksam in den später durchgeführten Eingriff eingewilligt [10]. Für diese Ansicht wird angeführt, dass die Bundesregierung in ihrem Entwurf des § 27b SGB V die Zweitmeinungsaufklärung ausdrücklich an § 630e BGB und den dort normierten Aufklärungspflichten orientiert [1].
  *Andererseits* wird auch die Ansicht vertreten, die *Zweitmeinungsaufklärung* sei eine *bloße Informationspflicht*, die dazu diene, den Anspruch der gesetzlich Versicherten auf Einholung einer Zweitmeinung zu realisieren [11]. Eine zusätzliche Aufklärungspflicht im Sinne des § 630e BGB solle nicht geschaffen werden, sobald also die dort normierten Aufklärungspflichten erfüllt seien, sei eine wirksame Einwilligung von Patient:innen bereits möglich und unabhängig von der Zweitmeinungsaufklärung [11]. Diese Ansicht geht davon aus, der Verweis im Regierungsentwurf zu § 27b SGB V auf § 630e BGB beziehe sich lediglich auf die Mündlichkeit und die Rechtzeitigkeit der Aufklärung [6]. Zudem stelle diese Haftungserweiterung eine unerwünschte Ungleichbehandlung von gesetzlich Versicherten und privat Versicherten dar, da nur für erstere die Haftung der Behandler:innen ausgeweitet würde [6].
  Zur Minimierung des Risikos, für Aufklärungsfehler zu haften, wie es der ersten zuvor dargestellten Rechtsansicht entspräche, sollte demnach in jedem Fall eine Aufklärung über das Recht auf Einholung einer Zweitmeinung erfolgen und entsprechend dokumentiert werden, um dies auch beweisen zu können.

Der Kreis der grundsätzlich zur Erbringung von Zweitmeinungen gemäß § 27b SGB V berechtigten Leistungserbringer:innen ergibt sich aus Absatz 3 der Norm und ist weit gefasst (Tab. 2). Das Zweitmeinungsverfahren weist hier eine Besonderheit auf. Taugliche Zweitmeiner:innen sind nämlich nicht nur zur Versorgung von gesetzlich Versicherten zugelassene Leistungserbringer:innen wie etwa Vertragsärzt:innen. Gemäß § 27b Abs. 3 Nr. 5 SGB V sind gerade auch nicht an der vertragsärztlichen Versorgung teilnehmende Ärzt:innen, die nur zum Zweck der Erbringung von Zweitmeinungen an der vertragsärztlichen Versorgung teilnehmen, taugliche Zweitmeiner:innen. Hierdurch sollen Privatärzt:innen dazu veranlasst werden, sich am Zweitmeinungsverfahren zu beteiligen [8]. Daneben sind Vertragsärzt:innen, Medizinische Versorgungszentren, ermächtigte Ärzt:innen und Einrichtungen und sowie Krankenhäuser gemäß § 27b Abs. 3 SGB V zur Erbringung einer Zweitmeinung berechtigt.

**Table 2:** 

Taugliche Zweitmeiner:innen	Genehmigung durch Kassenärztliche Vereinigung unter Voraussetzungen				
	Unabhängigkeit	Facharzttitel	Tätigkeitsdauer	Kenntnis aktueller Wissenschaft	
Zugelassene MVZ, Vertragsärzt:innen	Zweitmeinung nicht durch die indikationsstellenden Ärzt:innen oder Einrichtungen selbst	Innere Medizin und Kardiologie, Innere Medizin mit Schwerpunkt Kardiologie, Kinder- und Jugendmedizin mit Schwerpunkt Kinderkardiologie, Kinder- und Jugendmedizin mit Schwerpunkt Kinder- und Jugend-Kardiologie, bzgl. Implantationen von Herzschrittmachern oder Defibrillatoren auch Herzchirurgie	Seit mindestens fünf Jahren in der unmittelbaren Patientenversorgung tätig	Fortbildungsverpflichtung aus § 95d SGB V erfüllt	Entweder Weiterbildungsbefugnis oder akademische Lehrbefugnis
Zugelassene Krankenhäuser				Fortbildungsverpflichtung aus § 136b SGB V erfüllt	
Ermächtigte Ärzt:innen und Einrichtungen				Fortbildungsverpflichtung aus § 95d SGB V erfüllt	
Privatärzt:innen				Ausreichende Fortbildungspunkte	

Es ist hierbei stets zu berücksichtigen, dass *die Zweitmeinung NICHT durch die Ärzt:innen oder Einrichtungen erbracht werden darf, die den maßgeblichen Eingriff durchführen sollen*, § 27b Abs. 1 Satz 2 SGB V (Unabhängigkeit der Zweitmeiner:innen im Sinne des § 7 Abs. 5 Allgemeiner Teil Zm-RL). Hierdurch sollen finanzielle Anreize für eine bestätigende Zweitmeinung ausgeschlossen werden [12]. Ausgeschlossen sind laut Begründung der Bundesregierung zu § 27b SGB V neben den indikationsstellenden Ärzt:innen selbst auch dasselbe Krankenhaus, sowie Ärzt:innen aus derselben Berufsausübungsgemeinschaft oder Praxisgemeinschaft [1]. Dies lässt darauf schließen, dass auch Ärzt:innen, die in demselben Krankenhaus oder Medizinischen Versorgungszentrum angestellt sind wie die indikationsstellenden Ärzt:innen, aufgrund eines möglichen mittelbaren wirtschaftlichen Interesses als Zweitmeiner:innen ausscheiden [13]. Die Handhabung dieser Vorgabe im konkreten Einzelfall ist derzeit mangels einschlägiger Rechtsprechung nicht sicher vorhersehbar. Als Leitfaden kann angesichts von Sinn und Zweck des § 27b SGB V ausweislich der Gesetzesbegründung jedoch der folgende Gedanke dienen: Sobald Zweitmeiner:innen von der Durchführung des Eingriffs, der Gegenstand der Zweitmeinung ist, wirtschaftlich profitieren, ist ein Mangel an Unabhängigkeit zumindest naheliegend.

Bei Beantragung der Genehmigung zur Durchführung der Abrechnung von Zweitmeinungen müssen Zweitmeiner:innen zudem offenlegen, ob sie Interessenkonflikten aufgrund finanzieller Beziehungen unterliegen (§ 7 Allgemeiner Teil Zm-RL).

*Voraussetzung* der Durchführung der *Abrechnung* von Zweitmeinungsleistungen ist die Erteilung einer entsprechenden *Genehmigung* der regional zuständigen *Kassenärztlichen Vereinigung* (§ 7 Abs. 1 S. 2 Allgemeiner Teil Zm-RL). Dies gilt auch für in zugelassenen Krankenhäusern angestellte Ärzt:innen und Privatärzt:innen. Diese müssen zusätzlich bei der zuständigen Kassenärztlichen Vereinigung eine Ermächtigung beantragen. Die Genehmigung der Zweitmeinungserbringung ist abhängig von der Erfüllung bestimmter Voraussetzungen.

Die eingriffsspezifischen Anforderungen an Zweitmeiner:innen ergeben sich für die kathetergestützten elektrophysiologischen Herzuntersuchungen und Ablationen am Herzen aus § 2 Eingriff 7 Besonderer Teil Zm-RL. Demnach sind zur Erbringung der Zweitmeinungen Fachärzt:innen der Fachrichtungen Innere Medizin und Kardiologie, Innere Medizin mit Schwerpunkt Kardiologie, Kinder- und Jugendmedizin mit Schwerpunkt Kinderkardiologie oder Kinder- und Jugendmedizin mit Schwerpunkt Kinder- und Jugend-Kardiologie berechtigt.

Zur Erbringung von Zweitmeinungen bzgl. der Indikation zur Implantation eines Herzschrittmachers, eines Defibrillators oder eines CRT-Aggregats sind zusätzlich zu den bereits benannten Facharztgruppen auch Fachärzt:innen der Fachrichtung Herzchirurgie grundsätzlich berechtigt.

Aus den rechtlichen Grundlagen für die Erbringung der Zweitmeinungen ergibt sich nicht die Anforderung, dass Zweitmeiner:innen die Eingriffe tatsächlich in der ärztlichen Praxis selbst durchführen. Gegenstand des Zeitmeinungsverfahrens ist die Indikationsstellung. Diese kann grundsätzlich auch von Ärzt:innen geprüft werden, welche die Eingriffe nicht selbst durchführen.

Eingriffsübergreifend ergeben sich aus § 7 Allgemeiner Teil Zm-RL zudem grundlegende Anforderungen an Zweitmeiner:innen. Diese müssen eine besondere Expertise in dem jeweils maßgeblichen Fachgebiet aufweisen (vgl. auch § 27b Abs. 2 Satz 2 und 3 SGB V).

Für die kathetergestützten elektrophysiologischen Herzuntersuchungen und Ablationen am Herzen bedeutet dies zum einen, dass Zweitmeiner:innen eine der zuvor aufgeführten Facharztbezeichnungen aufweisen müssen. Nach Anerkennung einer solchen Facharztbezeichnung müssen die Zweitmeiner:innen für mindestens 5 Jahre (auf eine Vollzeittätigkeit bezogen, Teilzeittätigkeiten sind anteilig zu berechnen) in einem der maßgeblichen Fachgebiete unmittelbar patientenversorgend tätig gewesen sein (§ 7 Abs. 2 Satz 2 Allgemeiner Teil Zm-RL).

Zweitmeiner:innen müssen zum anderen auch Kenntnisse über den aktuellen Stand der Wissenschaft zu Diagnostik und Therapie (einschließlich Therapiealternativen) bzgl. des hier maßgeblichen elektrophysiologischen Eingriffs aufweisen. Diese Kenntnisse gelten als nachgewiesen, wenn die Fortbildungsverpflichtung aus § 95d SGB V (Vertragsärzt:innen) oder § 136b Abs. 1 Nr. 1 SGB V (Ärzt:innen in zugelassenen Krankenhäusern) erfüllt oder eine entsprechende Anzahl von der zuständigen Landesärztekammer anerkannter Fortbildungspunkte (Ärzt:innen, die nicht an der vertragsärztlichen Versorgung teilnehmen) erworben wurde, und entweder durch die zuständige Ärztekammer eine Weiterbildungsbefugnis erteilt oder eine akademische Lehrbefugnis verliehen wurde.

Bei Erfüllung aller vorgenannten Voraussetzung wird die Genehmigung zur Durchführung der Abrechnung von Zweitmeinungen durch die zuständige Kassenärztliche Vereinigung erteilt. Ärzt:innen, die nicht an der vertragsärztlichen Versorgung teilnehmen, werden für den Zeitraum, in dem sie am kassenärztlichen Zweitmeinungsverfahren teilnehmen, zur Durchführung der Abrechnung gemäß § 31 Abs. 2 Ärzte-Zulassungsverordnung i. V. m § 5 Abs. 2 Bundesmantelvertrag-Ärzte ermächtigt.

## Aufgaben der Zweitmeiner:innen

Die Pflicht zur Offenlegung von Interessenkonflikten trifft Zweitmeiner:innen auch im Verhältnis zu Patient:innen, die sie zwecks Einholung einer Zweitmeinung aufsuchen – allerdings nur auf Nachfrage. Die Offenlegung solcher finanziellen Beziehungen wie auch die Offenlegung von Interessenkonflikten hat zu Beginn einer Beratungsgesprächs zu erfolgen (§ 8 Abs. 2 Allgemeiner Teil Zm-RL).

Die Zweitmeiner:innen haben sodann die Patient:innen bzgl. des maßgeblichen Eingriff zu informieren und etwaige Therapiealternativen aufzuzeigen, so dass die Patient:innen in die Lage versetzt werden, die Notwendigkeit des geplanten Eingriffs zu beurteilen (§ 8 Abs. 1 Allgemeiner Teil Zm-RL). Die Beratung hat mündlich stattzufinden und soll Vorbefunde aus den Behandlungsunterlagen einbeziehen, wobei die Zweitmeiner:innen die Patient:innen auf aus ihrer Sicht fehlende oder unverwertbare Unterlagen hinzuweisen haben (§ 8 Abs. 4 und 5 Allgemeiner Teil Zm-RL).

Sofern die Patient:innen dies wünschen, teilen die Zweitmeiner:innen das Ergebnis des Zweitmeinungsverfahren entweder den indikationsstellenden Ärzt:innen mit oder erstellen einen zusammenfassenden ärztlichen Bericht, den sie den Patient:innen aushändigen (§ 8 Abs. 7 Allgemeiner Teil Zm-RL).

**Figure 1:**
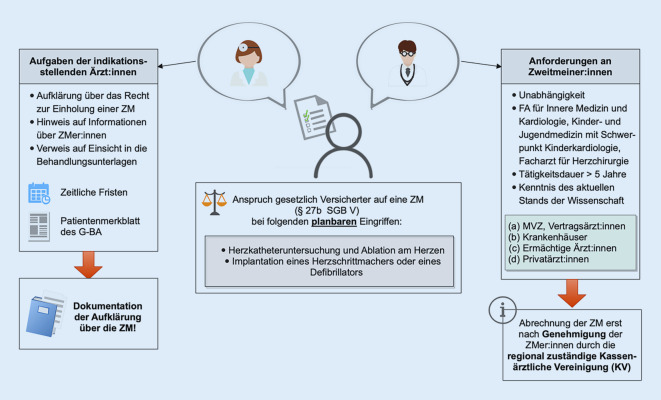

Eine Zusammenfassung der in diesem Artikel aufgeführten Aufgaben und Anforderungen des Zweitmeinungsverfahrens ist Abb. 1 zu entnehmen.

## Fazit für die Praxis

- In der ärztlichen Praxis ist eine ausreichende *Zweitmeinungsaufklärung* vorzunehmen und diese zu späteren Beweiszwecken adäquat zu *dokumentieren*.
- Durch Zweitmeiner:innen ist stets ihre *Unabhängigkeit* sicherzustellen.
- Aus dem Wortlaut des § 27b SGB V lässt sich lediglich entnehmen, dass eine Zweitmeinung „nicht bei einem Arzt oder einer Einrichtung eingeholt werden [kann], durch den oder durch die der Eingriff durchgeführt werden soll“.
- Die zuvor ausgeführte Erweiterung auf Ärzt:innen etwa derselben Berufsausübungsgemeinschaft ergibt sich aus den Gesetzgebungsmaterialien. Hier ist eine restriktive Handhabung empfehlenswert, so dass die Zweitmeiner:innen einer anderen unabhängigen Einrichtung zuzuordnen sein sollten.
- In Bezug auf die *Abrechnung* ist das *Genehmigungsverfahren* bei der jeweils zuständigen Kassenärztlichen Vereinigung einzuhalten. Grundsätzlich genehmigungsfähig ist die Zweitmeinungserbringung durch die o. g. Gruppen von Ärzt:innen.
- Die Abrechnung der Erbringung der Zweitmeinungen erfolgt nach den entsprechenden Regelungen des Einheitlichen Bewertungsmaßstabs.
